# Differential Actions of the Endocytic Collagen Receptor uPARAP/Endo180 and the Collagenase MMP-2 in Bone Homeostasis

**DOI:** 10.1371/journal.pone.0071261

**Published:** 2013-08-05

**Authors:** Daniel H. Madsen, Henrik J. Jürgensen, Signe Ingvarsen, Maria C. Melander, Reidar Albrechtsen, Andreas Hald, Kenn Holmbeck, Thomas H. Bugge, Niels Behrendt, Lars H. Engelholm

**Affiliations:** 1 The Finsen Laboratory, Rigshospitalet/Biotech Research and Innovation Centre, University of Copenhagen, Copenhagen, Denmark; 2 Department of Biomedical Sciences & Biotech Research and Innovation Centre, University of Copenhagen, Copenhagen, Denmark; 3 Matrix Metalloproteinase Unit, Craniofacial and Skeletal Diseases Branch, National Institute of Dental and Craniofacial Research, National Institutes of Health, Bethesda, Maryland, United States of America; 4 Proteases and Tissue Remodeling Section, National Institute of Dental and Craniofacial Research, National Institutes of Health, Bethesda, Maryland, United States of America; University of Bergen, Norway

## Abstract

A well-coordinated remodeling of uncalcified collagen matrices is a pre-requisite for bone development and homeostasis. Collagen turnover proceeds through different pathways, either involving extracellular reactions exclusively, or being dependent on endocytic processes. Extracellular collagen degradation requires the action of secreted or membrane attached collagenolytic proteases, whereas the alternative collagen degradation pathway proceeds intracellularly after receptor-mediated uptake and delivery to the lysosomes. In this study we have examined the functional interplay between the extracellular collagenase, MMP-2, and the endocytic collagen receptor, uPARAP, by generating mice with combined deficiency of both components. In both uPARAP-deficient and MMP-2-deficient adult mice the length of the tibia and femur was decreased, along with a reduced bone mineral density and trabecular bone quality. An additional decrease in bone length was observed when combining the two deficiencies, pointing to both components being important for the remodeling processes in long bone growth. In agreement with results found by others, a different effect of MMP-2 deficiency was observed in the distinct bone structures of the calvaria. These membranous bones were found to be thickened in MMP-2-deficient mice, an effect likely to be related to an accompanying defect in the canalicular system. Surprisingly, both of the latter defects in MMP-2-deficient mice were counteracted by concurrent uPARAP deficiency, demonstrating that the collagen receptor does not support the same matrix remodeling processes as the MMP in the growth of the skull. We conclude that both uPARAP and MMP-2 take part in matrix turnover processes important for bone growth. However, in some physiological situations, these two components do not support the same step in the growth process.

## Introduction

Remodeling of the extracellular matrix is required for a range of normal physiological processes but is also connected to a number of pathological conditions including arthritis, fibrosis and cancer [1], [2], [3], [4]. The most abundant protein constituents of the extracellular matrix are the collagens. Collagen type I is the principal component of bones and the dynamic formation and degradation of collagen type I are essential processes for both development and homeostasis of bones. Bone formation occurs through two distinct processes known as endochondral ossification and intramembranous ossification. Most bones of the body, such as the long bones, develop through initial superficial intramembranous ossification followed by endochondral ossification to establish the cancellous core of the bone. Endochondral ossification is dependent on vascular invasion into unmineralized cartilage, which is largely facilitated by osteoclasts [5]. Some flat bones of the skull on the other hand develop completely or partially through intramembranous ossification, which is characterized by bone formation directly within a soft connective tissue that is partially associated with a cartilage primordium [5], [6], [7]. During adulthood, bone remodeling serves to replace damaged tissue with new tissue, and to adapt to changes in the mechanical stress imposed on the bone.

Degradation of the extracellular matrix of the bones involves various types of osteogenic cell types and enzymes. Importantly, osteoblasts synthesize the collagenolytic matrix metalloproteinases (MMPs), MMP-2, MMP-13 and MT1-MMP (MMP14), and osteoclasts synthesize both MT1-MMP and the collagenolytic cysteine protease cathepsin K [8], [9]. In addition to collagenolysis in the pericellular environment mediated by extracellular proteases, a less studied intracellular pathway of collagen degradation also exists. This pathway involves the uptake of collagen and collagen-fragments by endocytic receptors, notably including uPARAP/Endo180 (hereafter designated uPARAP) [10], [11], [12], followed by degradation in the lysosomes [12], [13]. This pathway of collagen degradation has been shown to be active in fibroblasts, osteoblasts, chondrocytes, macrophages, sinusoidal hepatic endothelial cells and in a range of established mesenchymal cell lines [11], [14], [15], [16], [17], [18], [19], [20], [21].

Consistent with a role of uPARAP in matrix degradation, the expression of the protein *in vivo* is primarily observed at sites undergoing extensive tissue remodeling. These include pathological conditions such as cancer, fibrosis and osteoarthritis [17], [21], [22], [23], [24], [25], [26], [27], [28], [29], and under normal physiological conditions, expression has been observed in the developing lung and in the bones [18], [30]. In addition, uPARAP has been found to be functionally involved in protection against tissue fibrosis [21], [28], [29] and in promotion of breast tumor progression [24], [31] and possibly glioma invasion [25].

During osteogenesis in mouse embryos a pronounced level of uPARAP expression has been demonstrated using *in situ* hybridization and immunohistochemistry, with expression mainly restricted to osteoblasts [16]. In accordance with this expression pattern, newborn uPARAP-deficient mice display a 6–8% reduction in bone length [18]. This reduction, however, appeared to be transient with no significant difference in bone length observed for 8-day old mice [18]. The role of uPARAP in bone apposition and skeletal homeostasis in adult mice has not been studied. A recent study of a severe bone disease affecting certain strains of cattle further supports a role of uPARAP in bone development. The underlying cause of this disease, known as the crooked tail syndrome, was found to be an inactivating point-mutation in the *MRC2* gene encoding uPARAP [32]. This syndrome leads to dramatic bone defects, ultimately necessitating the euthanasia of the affected animals.

A central question relates to the redundancy and functional interplay between uPARAP and collagenolytic enzymes, such as the MT1-MMP – MMP-2 axis. In bone development, MT1-MMP serves to directly degrade collagen [7] but also to activate pro-MMP-2 (Gelatinase-A) [33], [34]. MMP-2 is another collagenolytic protease, which is highly expressed in osteocytes and osteoblasts [35], [36]. In humans, mutations in the gene encoding MMP-2 cause severe bone disorders in humans known as multicentric osteolysis with arthopathy [37], [38], whereas MMP-2-deficient mice display only minor impairments in bone development [39]. However, more detailed analyses of MMP-2-deficient mice have recently revealed that many of the characteristics of the human disorder are in fact recapitulated in these mice [36], [40].

We have previously demonstrated in cell culture that defined collagen fragments released by initial cleavage of native collagen fibers serve as preferred substrates for uPARAP mediated uptake and degradation [12]. Such defined collagen fragments are also susceptible to further degradation by MMP-2 [41], [42], suggesting that uPARAP and MMP-2 participate in parallel steps of collagen degradation. Indeed, indications of a functional overlap between the two proteins have recently been found *in vitro*, where siRNA mediated downregulation of uPARAP led to a compensational increase in cellular MMP-2 activity [43].

Intriguingly, it was discovered that combined deficiency of uPARAP and MT1-MMP in mice leads to dramatically impaired bone formation and is incompatible with postnatal survival [18]. In this study, however, it was not investigated whether the observed defects were a direct consequence of concurrent loss of uPARAP and MT1-MMP collagenolytic activity, or a consequence of uPARAP deficiency combined with impaired pro-MMP-2 activation. To address the putative functional overlap between uPARAP and MMP-2 we have now studied the effects of uPARAP deficiency, MMP-2 deficiency and combined uPARAP and MMP-2 deficiency in mice, with special emphasis on bone development and homeostasis.

## Materials and Methods

### Breeding and Generation of Gene-targeted Mice

Mice deficient of uPARAP and mice deficient of MMP-2 have been described previously ([11] and [39], respectively). Before the start of the study, uPARAP-deficient mice were backcrossed for 23 generations and MMP-2-deficient mice for 21 generations into an FVB/N background. Heterozygous FVB/N uPARAP (U+/−) mice were mated with heterozygous FVB/N MMP-2 (M+/−) mice to generate F1 mice. The double heterozygous (U+/−, M+/−) mice were interbred to generate F2 mice. The following breeding pairs were set up in order to generate F3 mice that were used in this study: U+/−, M+/− mice were mated with U−/−, M−/− mice, or U+/−, M−/− mice were mated with U−/−, M+/− mice. This breeding program resulted in equal numbers of the following littermate mice: U+/−, M+/− (U+M+) mice, U−/−, M+/− (U−M+) mice, U+/−, M−/− (U+M−) mice and U−/−, M−/− (U−M−) mice. All pups were weaned at 21–28 days of age and genotyped as described below. At 33 weeks of age, the mice were euthanized under anesthesia and perfused with PBS, followed by 4% paraformaldehyde (PFA). All animal experiments were conducted according to institutional guidelines and approved by the Danish Animal Experiments Inspectorate.

### Genotyping

DNA from a 1–2 mm tail tip biopsy was isolated according to a standard protocol. uPARAP and MMP-2 genotypes were determined by individual PCR analyses using the indicated primers. For uPARAP, the wildtype allele was amplified using the following primer set: (Exon3-5′; TCTACACCATCCAGGGAAACTCAC, Exon3-3′; TTAAACTGGTAACAGCTGTCAGTC), and the targeted allele using the primer set (uPARAP targ1; TCCTACAAATACACGCTGGCGATA and uPARAP targ2; GCAGTTCCCTTTTAAATGCAAATCA) (11). For MMP-2, the wildtype allele was amplified using the primer set (MMP-2-5′; CAACGATGGAGGCACGAGTG, MMP-2-3′; GCCGGGGAACTTGATCATGG), and the targeted allele using the primer set (MMP-2 targ1; ATGATTGAACAAGATGGATTGCAC, MMP-2 targ2; TTCGTCCAGATCATCCTGATCGAC) (27,32). The reaction conditions for both uPARAP and MMP-2 PCRs were: 95°C for 15 min, followed by 34 cycles of 94°C for 45 s, 60°C for 45 s and 72°C for 90 s. The PCR reactions were terminated by a final extension at 72°C for 10 min. All genotypes were confirmed by repeated PCR analyses at the termination of the experiment.

### Isolation and Preparation of Bones

Following perfusion fixation, the left leg (tibia and femur) and the calvaria were dissected and all associated soft tissue was thoroughly removed. The bones were fixed in 4% PFA for 48 hours at 4°C, after which they were used for determination of bone mineral density (µCT analysis or PIXImus densitometer analysis) and long bone length (see below). The bones were then decalcified in 10% EDTA, pH 7.4 under microwave irradiation at 50°C for 2 hours using a MicroMED TT/Mega microwave oven (Milestone, Bergamo, Italy). Fresh EDTA solution was added and the bones were incubated at 4°C for 3 days. Bones were rinsed in tap water, which was then replaced with 70% ethanol. The calvariae were then bisected perpendicular to the sagittal suture through the center of the parietal bones. These calvarial specimens were finally embedded in paraffin to allow sectioning for histology from the central part of the bone.

### Western Blotting

Organs from a 30 weeks old female uPARAP-deficient mouse and a littermate female wildtype mouse were isolated after perfusion of the mice with 10 ml PBS as described above. The organs were frozen immediately and subsequently ground into a fine powder in liquid nitrogen using a mortar and pestle. This material was homogenized in a CHAPS lysis buffer (0.1 M Tris-HCl, pH 7.5, 50 mM NaCl, 2% CHAPS, 0.5% v/v protease inhibitor cocktail III (Calbiochem)), using a polytron homogenizer. The organ homogenates were clarified by centrifugation at 20,000×g for 15 min at 4°C. Protein concentrations were determined using a BCA assay according to the manufacturer’s instructions (Pierce, Rockford, IL). SDS-PAGE was performed with 30 µg of organ homogenate protein loaded in each well and was followed by electroblotting onto PVDF membranes. For detection of uPARAP, blots were incubated with 20 ng/ml of ^125^I−labeled anti-uPARAP antibody 2h9F12 as previously described [12], followed by phosphorimager analysis.

### Bone Length Measurements

Tibiae and femora from approximately equal numbers of male and female mice of each of the genotypes were measured, using a sliding gauge, by four researchers who were unaware of the mouse genotypes. For each bone, the average of the four measurements was used for subsequent data analysis.

### Bone Density Measurements

Femora from female mice were examined by µCT analysis, using an eXplore Locus SP µCT scanner (GE Medical Systems, London, Ontario, Canada) at 8-µm resolution. Reconstructed 3D volumes of the distal femora were analyzed using Microview software (GE Medical Systems). To analyze the trabecular bone, a region of interest (ROI) was defined by an elliptical cylinder placed in the center of the bone at a fixed relative distance (17% of the total length) from the distal end of the femur. To analyze the cortical bone mineral density (BMD), a ROI encompassing the entire perimeter of the bone structure was placed at the same location from the distal end of the femur as the elliptical cylinder. All comparative analyses were performed with fixed thresholds. For the cortical BMD measurements a high threshold was chosen to exclude almost all of the trabecular bone. In each scan four bones (one bone of each genotype) were included, allowing the measurements in each scan to be normalized to the U+M+ bone. Using a PIXImus dual-energy X-ray densitometer (Lunar Corp., Madison, WI), bone mineral accumulation (BMA) values, expressed as mass per surface area, were measured for the dissected calvariae in duplicates. This composite measure of specimen thickness and density was performed along with separate measurements of thickness alone. For these BMA measurements, a low threshold value for bone recognition had to be selected to enable the software to recognize the thinnest parts of the calvaria. A defined region of ROI of 39×30 pixels was chosen and placed in the center of the parietal bone of each calvaria.

### Histological Examination of Bones

Calvariae were sectioned at 3 µm on a Leica SM2000R microtome followed by hematoxylin and eosin staining. Using an Olympus BX51 microscope and Visiopharm image software (Visiopharm, Hørsholm, Denmark), stitched superimages were created and the thickness of the calvaria was measured 3 mm to each side from the sagittal suture. For the quantification of the fraction of empty lacunae, the number of empty and filled lacunae were counted manually, starting at a distance 2 mm from the sagittal suture and moving outwards, while counting empty and filled lacunae until the end of the bone was reached or more than 100 lacunae counted. This procedure was repeated for the other half of the bisected calvarial bone. For the examination of osteocyte apoptosis an experienced pathologist (R. A.), unaware of the genotypes, identified apoptotic cells by the presence of apoptotic bodies characterized by chromatin condensation and nuclear fragmentations [44]. The frequency of apoptotic osteocytes was determined by manual counting of cells with visible apoptotic bodies among 200 osteocytes (distributed on two cross sections from each calvaria, 100 cells on each section).

### Statistical Analyses

Differences in the weight curves for mice of the various genotypes were analyzed using a general linear model with repeated measures (PROC MIXED, SAS v9.2). Binary variables for the two first and the two last time points were included in order to adjust for non-linearity. Model validation was done using conventional methods. Analysis of differences in BMAs, bone lengths or fraction of empty lacunae was carried out by pairwise analysis using two-tailed Student´s t-test. Analysis of differences in BMDs, trabecular thickness (Tb.Th.) or trabecular spacing (Tb.Sp.) was carried out by pairwise analysis using a paired two-tailed Student´s t-test. P values below 5% were considered significant.

## Results

### uPARAP Expression in Adult Mice

**Figure 1:**
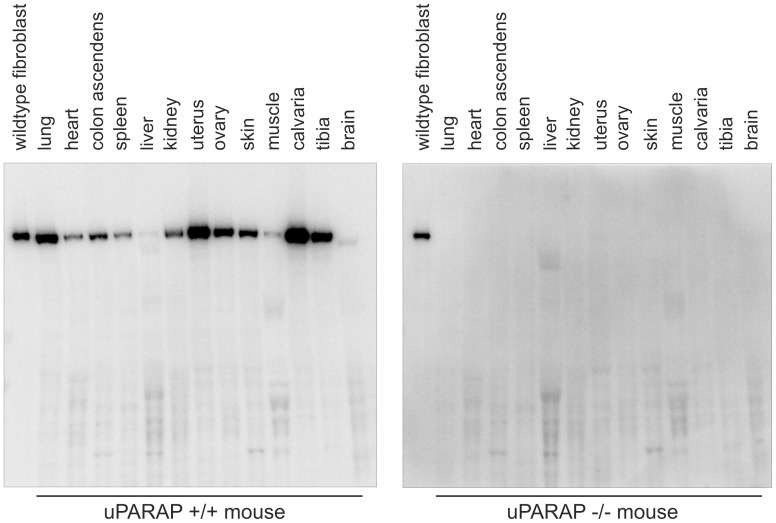
Expression of uPARAP in various mouse organs. Western blot analysis showing the expression of uPARAP in a number of different organs collected from a female wild-type mouse (left panel). Organs from a littermate uPARAP −/− mouse were analyzed as a control for antibody specificity (right panel). Equal protein amounts were loaded in each well. In each panel, a lysate of wildtype mouse fibroblasts was included as a positive control (left lane on each blot).

**Figure 2:**
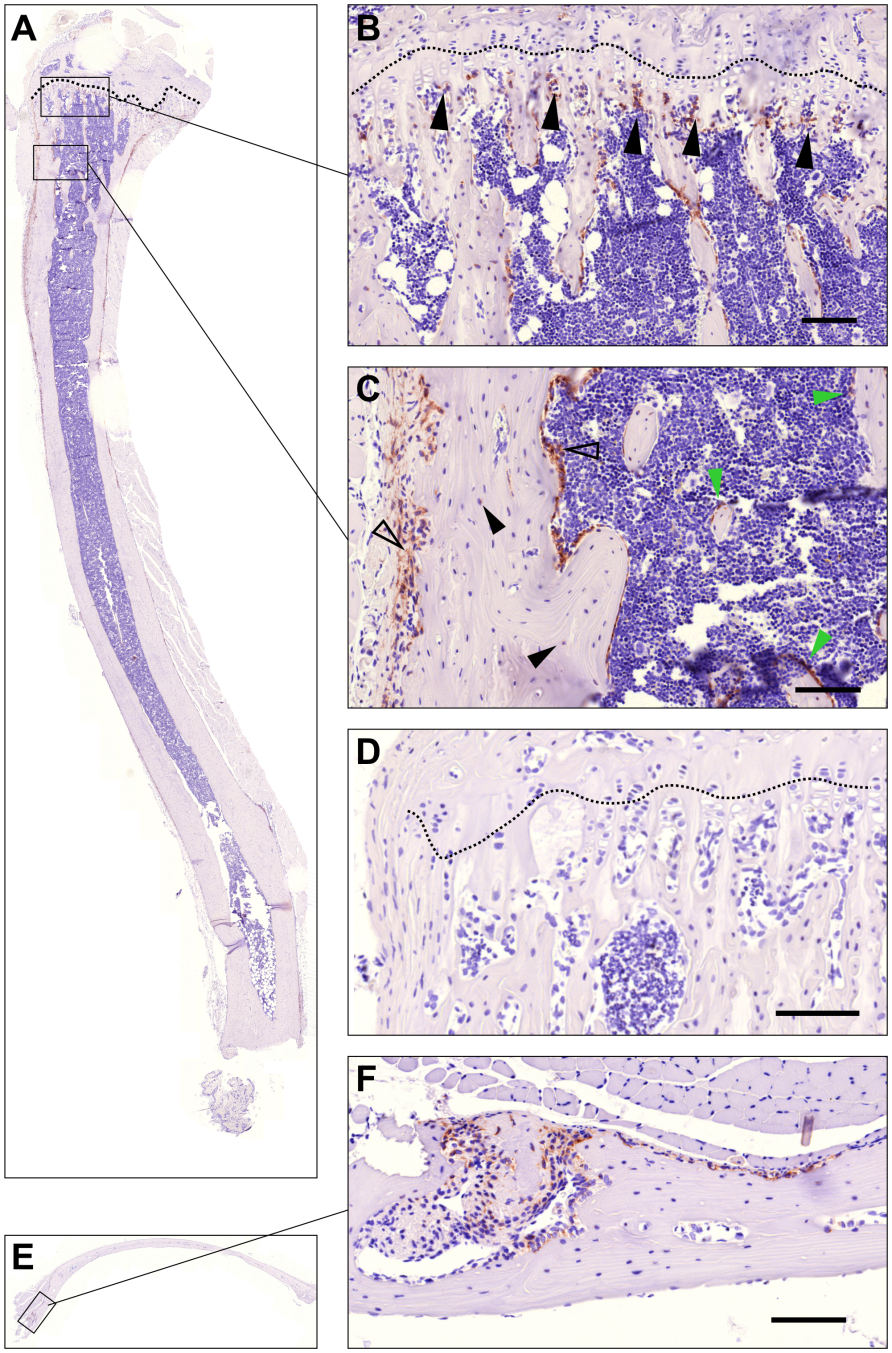
uPARAP in bone growth and homeostasis in adult mice. The expression of uPARAP in long bones visualized by IHC staining. In saggital sections of proximal tibiae from 14 weeks old mice (panel A–C), uPARAP positive osteogenic cells (panel B, black arrowheads) populate the surfaces of nascent bone trabeculae and cartilage cores of the primary spongiosa (zone of hypertrophy indicated by dotted line in A, B and D). In addition to the primary spongiosa, uPARAP is also expressed by osteoblastic cells lining (panel C) both cortical (open arrow heads) and cancellous bone (green arrow heads). Scattered osteocytes (black arrow heads) entrapped in the cortical bone also express uPARAP. Negative control tibiae isolated from uPARAP-deficient mice (D) were negative for uPARAP staining, confirming specificity of the antibody. In calvariae (panel E and F) uPARAP expression was detected in osteogenic cells populating the suture and endosteum. Bars: 100 µm.

During mouse embryogenesis, a strong expression of uPARAP has been demonstrated in the bones at sites of ossification [16] and in newborn mice, uPARAP expression is likewise observed in the developing bones [18]. In adult mice and in humans, an early analysis of uPARAP mRNA revealed expression in a variety of tissues [45] but the relative expression of uPARAP in bone compared to soft tissue has not been studied. To address this, uPARAP expression in tissue homogenates of a panel of organs, including tibia and calvaria, from adult mice were analyzed by Western blotting (Fig. 1). uPARAP expression was seen in all tested organs although at very low levels in liver, muscle and brain. Calvaria and tibia, along with lung and uterus, were the organs with the highest level of uPARAP expression. To investigate the expression pattern of uPARAP in adult mouse bones in more detail, immunohistochemical analyses were carried out. In both the long bones and the calvaria, a pronounced expression of uPARAP was noted (Fig. 2). In the tibia a strong expression was observed in osteogenic cells in the area of active bone formation (Fig. 2A–B). In addition, osteoblastic bone lining cells in the periosteum, the endosteum, as well as on the trabecular structures, were uPARAP positive, along with scattered osteocytes in the cortex (Fig. 2C). In the calvaria a strong expression was also observed in osteogenic cells within the cranial sutures (Fig. 2E–F). To ensure staining specificity, bones from uPARAP-deficient littermates were included and were in all cases negative (Fig. 2D). Thus, expression of uPARAP in adult bone included both sites of active growth (the long bone growth plate and cranial sutures) and areas important for bone homeostasis (the lining cells involved in continuous bone remodeling [46]). These analyses open the possibility that uPARAP is involved in collagen remodeling processes in bones, also in the adult mouse.

### Double Deficiency for uPARAP and MMP-2 has Limited Consequences for Mouse Development

Since previous studies have revealed a dramatic phenotypic effect of uPARAP and MT1-MMP double deficiency [18], we wanted to investigate whether this was related to the function of MT1-MMP in activating the MMP-2 proenzyme. Consequently, we generated mice with combined uPARAP and MMP-2 deficiency. To acquire the relevant groups of mice, we interbred FVB/N mice with targeted uPARAP or MMP-2 alleles to generate littermate mice with either uPARAP deficiency (U−M+), MMP-2 deficiency (U+M−) or combined uPARAP and MMP-2 deficiency (U−M−), as well as littermate uPARAP and MMP-2 expressing control mice (U+M+). The four groups of mice were born in the expected Mendelian ratio and somewhat surprisingly, upon gross inspection the U−M− mice were indistinguishable from their uPARAP and MMP-2 expressing littermates (results not shown). MMP-2-deficient mice have previously been reported to be significantly smaller than wildtype mice [39]. To evaluate if uPARAP further affected the overall growth a cohort study was established wherein the mice were weighed once a week from weaning until the age of 16 weeks (Fig S1). We found that U−M+ mice were not significantly different from U+M+ mice whereas U+M− mice were significantly smaller. U−M− mice were not significantly different from the U+M− mice. This situation was unchanged for 33 weeks old mice (data not shown).

### uPARAP and MMP-2 Deficiency Reduce Length and Density of Long Bones

**Figure 3:**
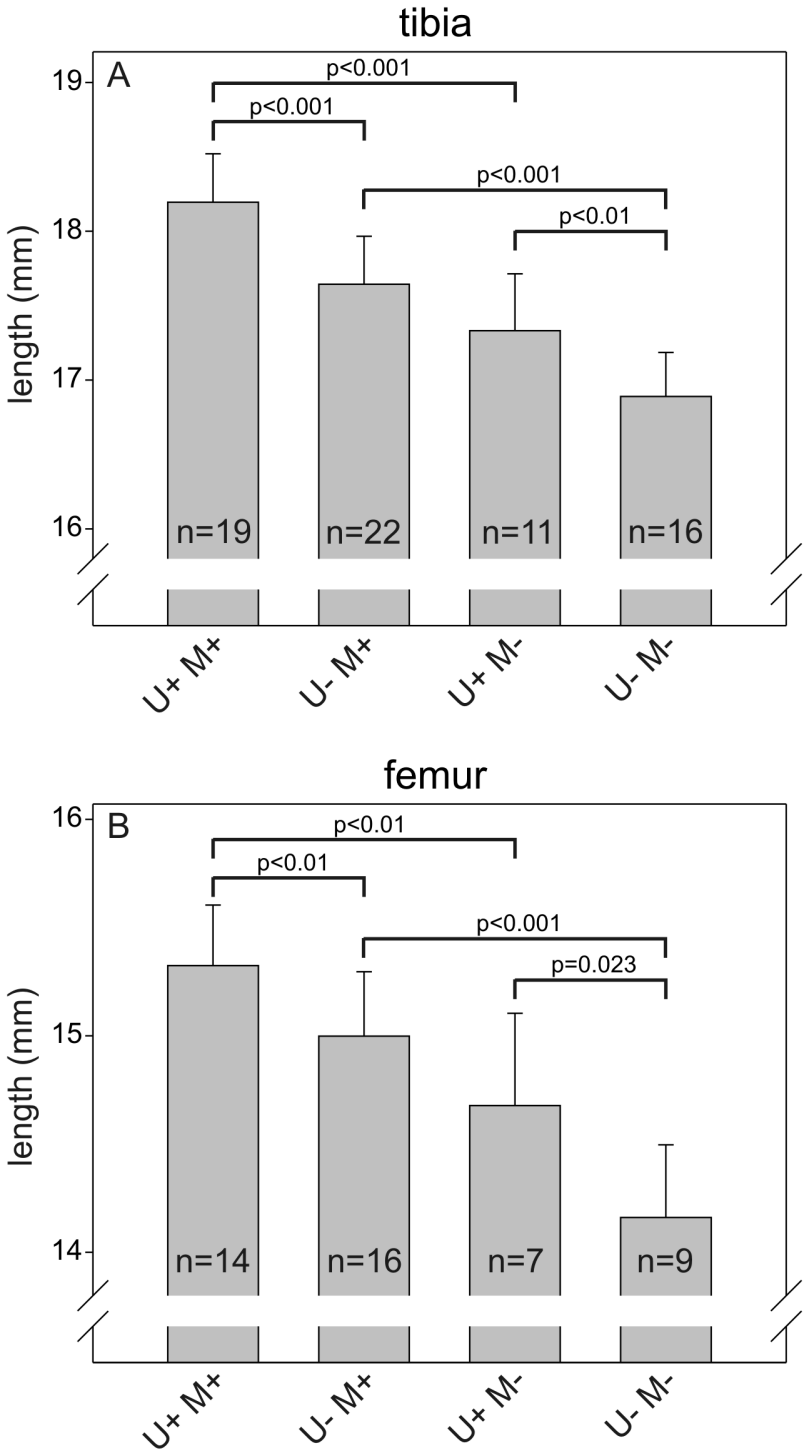
Single and combined deficiency of uPARAP and MMP-2 results in reduced growth of long bones. The lengths of tibiae (A) and femora (B) from 33 weeks old mice with the indicated genotypes were measured after dissection. Error bars: SD. n = number of mice with indicated genotype.

MMP-2 deficiency has been reported to result in reduced length and density of the long bones in adult mice [36], [40], [47]. The role of uPARAP deficiency has not been examined in the adult organism, but newborn uPARAP-deficient mice also display a small reduction in the bone length [18]. This opened the possibility that a functional overlap could exist between these collagen processing proteins, specifically in bone growth, and that such an overlap could result in a more severe impairment of bone development in double-deficient mice compared to mice with single-deficiency of either uPARAP or MMP-2. To investigate this, we measured the length of the femur and tibia from adult mice with the same designations as above (Fig. 3). As expected, U+M− mice displayed reduced length of both types of long bones compared to U+M+ mice (4.7% and 4.2% reduction for tibia and femur, respectively). U−M+ mice also had shorter bones than U+M+ mice (3.0% and 2.1% reduction for tibia and femur, respectively), demonstrating that the bone growth impairment caused by this deficiency is indeed reflected in adult life. Furthermore, U−M− mice displayed a larger decrease in bone length than mice with the single deficiencies (7.3% and 7.7% reduction for tibia and femur, respectively, compared to U+M+). All of the observed differences were statistically significant.

**Figure 4:**
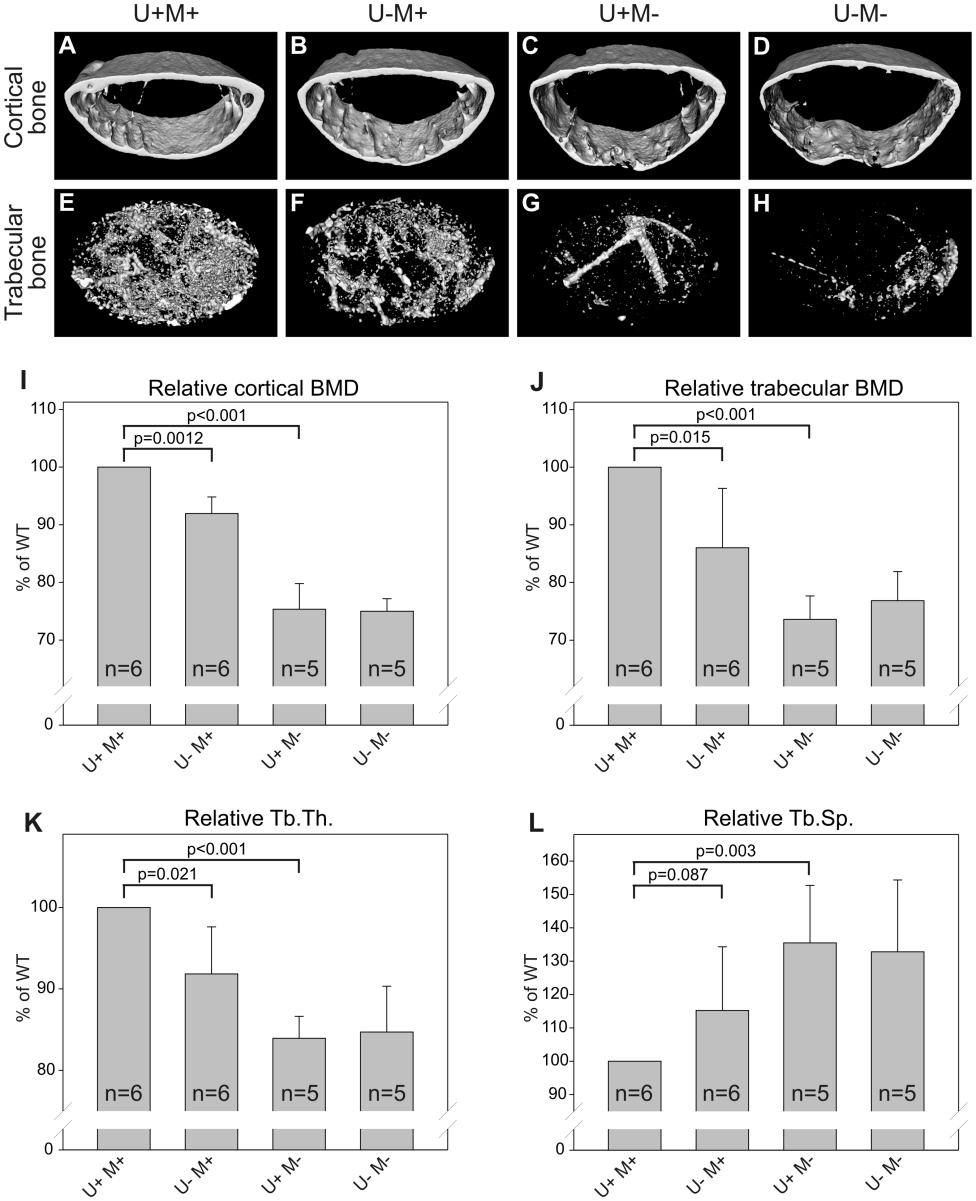
MMP-2 deficiency leads to reduced bone density and compromised structural quality of long bones. µCT isosurface renditions of the distal femora from 33-weeks old mice (A–H) thresholded for either the cortex (A–D), or the trabecular bone (E–H) (see Methods). Relative cortical BMD (I), relative trabecular BMD (J), as well as trabecular thickness (K) and trabecular spacing (L) was determined using the rendered volumes. Error bars: SD. n = number of mice with indicated genotype.

We next examined the bone mineral density (BMD) as well as the trabecular bone structure of the femora, using high resolution µCT analysis (Fig. 4). This analysis demonstrated that single-deficiency of either uPARAP or MMP-2 led to a reduced BMD in the adult mouse (Fig. 4I–J). Both a reduction in the cortical BMD (Fig. 4A–D and Fig. 4I) and in the trabecular BMD (Fig. 4E–H and Fig. 4J) were observed. Somewhat surprisingly, we did not observe any additive effect for the mice with combined uPARAP and MMP-2 deficiency. Based on µCT 3D reconstruction of femora, the quality of the trabecular bone was further analyzed. In each of the single-deficient mice trabecular thickness was reduced (Fig. 4K) and accordingly the trabecular spacing was increased (Fig. 4L), although the increase observed for the uPARAP-deficient mice was not statistically significant (p = 0.087). Thus, each of the single-deficiencies appeared to compromise the microstructural quality of the trabecular bone. However, in accordance with the BMD analyses we observed no additive effect for the double-deficient mice (Fig. 4K–L).

### uPARAP and MMP-2 have Opposite Effects on Bone Formation in the Flat Bones of the Skull

**Figure 5:**
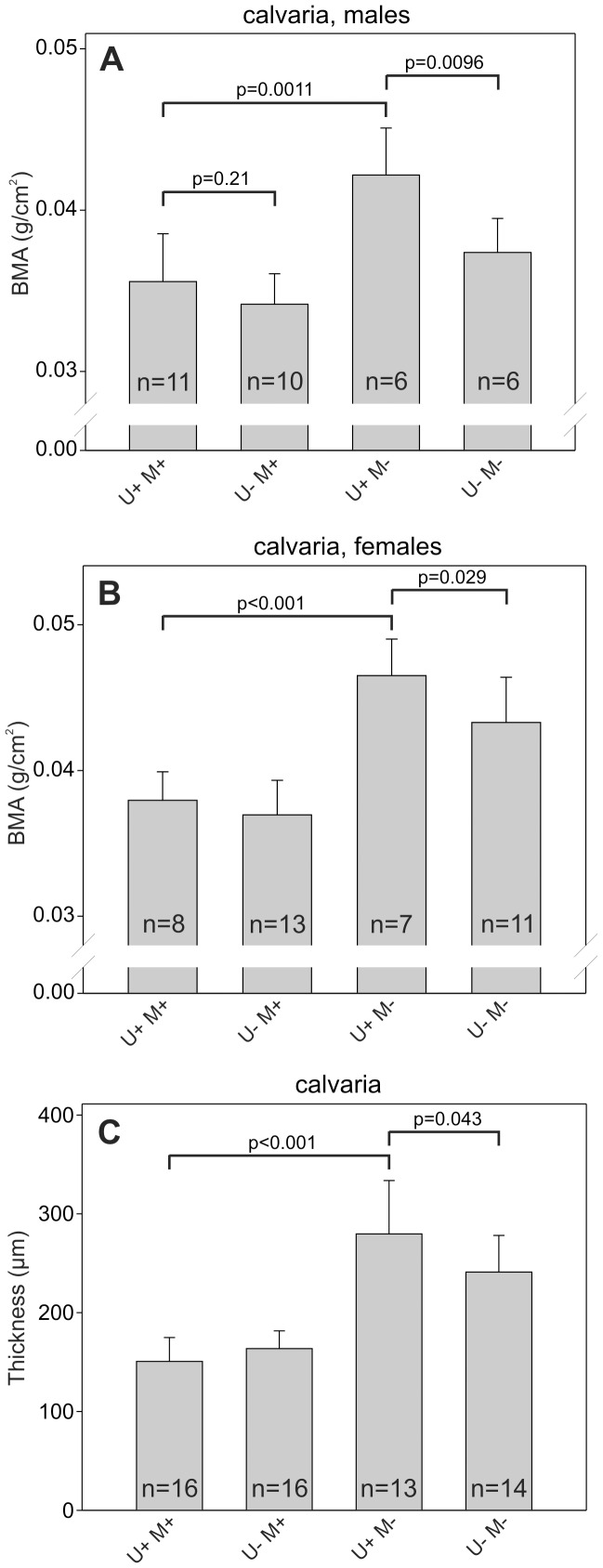
Superposition of uPARAP deficiency on MMP-2 deficiency counteracts the calvarial phenotypes of MMP-2-deficient mice. The BMA of the central part of the calvariae from 33 weeks old male (A) or female (B) mice of the indicated genotypes was measured. The thickness of the calvariae from 33 weeks mice (C) was measured using Visiopharm software. Error bars: SD. n = number of mice with indicated genotype.

In contrast to the long bones several flat bones of the skull develop exclusively through intramembranous ossification. To evaluate the influence of uPARAP and MMP-2 on bone homeostasis in the calvaria we measured the bone mineral accumulation (BMA) of the bones using an x-ray densitometer (Fig. 5A and B). We observed a marked increase in BMA of adult U+M− mice compared to U+M+ mice but no difference between U−M+ mice and U+M+ mice. Surprisingly, however, U−M− mice displayed a significantly reduced calvarial BMA compared to the U+M− mice (Fig 5A and B, column 3 and 4). Since the BMA measurements are dependent on both the mineral density and the thickness of the bone, we went on to measure the thickness of the calvariae (Fig. 5C and D). This analysis showed that the thickness of the calvariae of the different genotypes followed the same pattern as observed with the BMA measurements. In accordance with previously reported observations (24), we thus found a marked increase in the thickness of calvariae of U+M− mice compared to U+M+ mice (Fig. 5C, column 1 and 3). Furthermore, we observed a reduced thickness of the calvariae of U−M− mice compared to U+M− mice (Fig. 5C, column 3 and 4).

### uPARAP and MMP-2 Affect Development of the Calvarial Canalicular System

**Figure 6:**
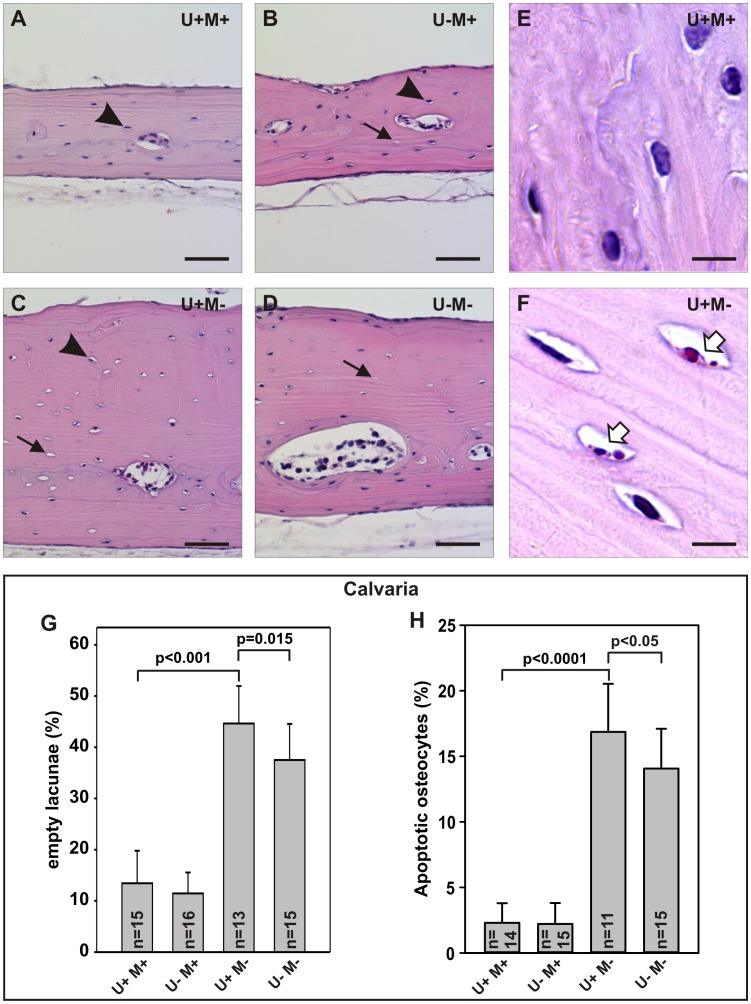
Combined deficiency of uPARAP and MMP-2 reduces the osteocytic death resulting from MMP-2 deficiency. H&E stained calvariae from mice of the indicated genotypes at low magnification (A–D) and high magnification (E–F). Empty and filled lacunae are exemplified with arrows and arrowheads, respectively (A–D). Apoptotic cells as determined by the observation of apoptotic bodies are exemplified with white arrows (F). These calvarial sections were also utilized for the measurements of thickness, illustrated quantitively in Fig. 5C. Scale bar in A–D: 50 µm, E–F: 8 µm. The fraction of empty lacunae in the mouse calvariae was quantified by counting approximately 200 lacunae from each calvaria (G). The frequency of apoptotic osteocytes was quantified by counting the number of cells with visible apoptotic bodies among 200 osteocytes from each calvaria (H). Error bars: SD. n = number of mice with indicated genotype.

The increased thickness of the calvariae in the adult MMP-2-deficient mice has previously been suggested to be a likely secondary effect of a disrupted canalicular system [36]. Such a defect leads to increased osteocytic apoptosis, leaving a high number of empty lacunae in the bone [36], [48]. Therefore, one possible explanation for the effects of combined uPARAP and MMP-2 deficiency on the calvarial thickness and density could be that uPARAP deficiency counteracts the impairment of canalicular development seen in MMP-2-deficient mice. To test this hypothesis, we examined the extent of osteocytic death through quantification of empty lacunae in calvariae of mice of the four different genotypes (Fig. 6). As expected, we observed a dramatic increase in the fraction of empty lacunae in the U+M− mice compared to U+M+ mice (Fig. 6C, column 1 and 3). In contrast, we did not find any difference in the fraction of empty lacunae in U−M+ mice compared to U+M+ mice (Fig. 6C, column 1 and 2). Interestingly, however, we did observe a reduction in the fraction of empty lacunae in U−M− mice compared to U+M− mice (Fig. 6C, column 3 and 4). We next performed a direct examination of calvarial osteocyte apoptosis in mice of the four different genotypes (Examples shown in Fig. 6E–F). In line with the analysis of empty lacunae, we found a dramatic increase in the fraction of apoptotic osteocytes in U+M− mice compared to U+M+ mice (Fig 6H, column 1 and 3) and a reduction in the fraction of apoptotic osteocytes in U−M− mice compared to U+M− mice (Fig 6H, column 3 and 4). These findings suggest that uPARAP deficiency opposes the increase in osteocytic death observed in the MMP-2-deficient mice.

## Discussion

Even though an intracellular pathway of collagen degradation was proposed almost four decades ago [49], [50], little is known about the functional role of this pathway in physiological conditions. In contrast, a substantial knowledge has accumulated regarding the extracellular degradation pathway, which proceeds through the action of secreted or membrane-attached proteases [4], [51].

Since uPARAP is expressed in bone and is a major player in the first-mentioned pathway [11], [29], we wished to examine the long-term consequences of uPARAP deficiency for bone homeostasis, alone or in combination with MMP-2 deficiency. We demonstrated that mice with combined deficiency of uPARAP and MMP-2 were perfectly capable of postnatal survival and displayed an overall normal growth and development. This is remarkable in light of the severe developmental defects found in mice with combined deficiency for uPARAP and the MMP-2 activating enzyme, MT1-MMP [18]. Thus, our study reveals that the latter defects were not, or only to a minor degree, a consequence of impaired pro-MMP-2 activation but must be ascribed to the absence of other functions of MT1-MMP, such as direct collagen cleavage.

At this point, we initiated a detailed analysis of the hard tissue of uPARAP and MMP-2-deficient mice, focusing initially on the long bones. In adult, unchallenged mice, no phenotypic abnormality has so far been reported to result from uPARAP deficiency alone. However, in this study we found that adult uPARAP-deficient mice have reduced length of the femur and tibia. This was also the case in mice deficient of MMP-2 alone. In mice with combined uPARAP and MMP-2 deficiency, we observed a further decrease in the bone lengths, which could point to a functional overlap between uPARAP and MMP-2 in long bone growth. A detailed µCT based analysis of the femoral bone structure demonstrated that both uPARAP and MMP-2 single-deficiency led to a reduction in the BMD as well as a reduced quality of the trabecular bone. Since these parameters are known to reflect the strength of the bone, our study thereby demonstrates an additional function of uPARAP in the maintenance of bone strength. However, when combining uPARAP and MMP-2 deficiency, no additive effect was observed in these analyses and that the complete pattern was even more complicated was shown by studies on the cranium as discussed below.

One of the most striking phenotypes of MMP-2-deficient mice is the altered composition of the flat bones of the calvaria [36], [40]. As MMP-2-deficient mice age, they develop calvarial bones with increased thickness compared to wildtype mice, indicating that MMP-2 activity is required for proper bone homeostasis. In this study we were able to confirm these effects of MMP-2 deficiency, but we did not, observe any effect of uPARAP single-deficiency on either thickness or bone mineral accumulation (BMA) of the calvaria of adult mice. This is consistent with previous examinations of newborn uPARAP-deficient mice where no impact on calvarial bone mineral density was observed [18]. Very surprisingly, however, we observed a reduction of both thickness and BMA in mice with combined uPARAP and MMP-2 deficiency compared to the MMP-2 single-deficient mice. Considering that the measured thickness follows the same pattern as the BMA measurements suggests that the effects on the calvarial BMA are primarily caused by changes in the calvarial thickness and not by changes in the mineral density.

The calvarial phenotype of MMP-2-deficient mice has been suggested to be a result of an observed disruption of the canalicular system and a consequent increase in osteocytic death, leaving a substantial fraction of the lacunae empty [36]. Excessive osteocytic death has also been observed in mice carrying a targeted mutation in *col1a1*, which results in the formation of collagen type 1 that is resistant to MMP-mediated remodeling [48]. As reported by Inoue *et al.* [36], we found that MMP-2 deficient mice display a dramatic increase in the fraction of empty lacunae in the calvaria. We also observed a dramatic increase in the frequency of apoptotic osteocytes in MMP-2 deficient mice. This finding indicates that osteocyte apoptosis is the likely cause of the increase in empty lacunae and the presence of apoptotic cells suggests that the observed phenotypes could be exacerbated if the mice were allowed to age further. The increased osteocyte apoptosis in the MMP-2 deficient mice is likely a consequence of a disturbed lacuna-canalicular network due to impaired matrix remodeling in these tissue compartments. Examination of the effect of uPARAP deficiency on osteocytic death strikingly revealed a reduction in empty lacunae as well as apoptotic osteocytes in the mice with combined deficiency of uPARAP and MMP-2, compared to the MMP-2 single-deficient mice. These observations demonstrate that uPARAP-deficiency partially rescues the calvarial phenotypes that are observed in the MMP-2 deficient mice in terms of calvarial thickness, osteocyte absence in lacunae, and osteocyte apoptosis.

Altogether, our work emphasizes the importance of uPARAP and intracellular collagen degradation in bone development and homeostasis. Importantly, uPARAP can either support or counteract the physiological processes that involve MMP-2 activity, depending on the bone compartment. This finding indicates that the interplay with extracellular proteases in physiological conditions could be more complex than previously assumed.

## Supporting Information

Figure S1 **Post-weaning growth of mice with single and combined deficiency of uPARAP and MMP-2.** The weight of a prospective cohort of mice was measured once a week after weaning for 13 weeks. The weight curves were compared using a two-way repeated measures ANOVA (see Methods). This analysis showed that the mice separated into two groups with U+M+ and U−M+ mice having a significantly faster weight gain than U+M− and U−M− mice.
(PDF)
