# Supplementary figures and images for: Differential Actions of the Endocytic Collagen Receptor uPARAP/Endo180 and the Collagenase MMP-2 in Bone Homeostasis
Source: PLoS One. 2013 Aug 5;8(8):e71261. doi: 10.1371/journal.pone.0071261 (PMC3734290; doi:10.1371/journal.pone.0071261)

Supplemental Fig. 1

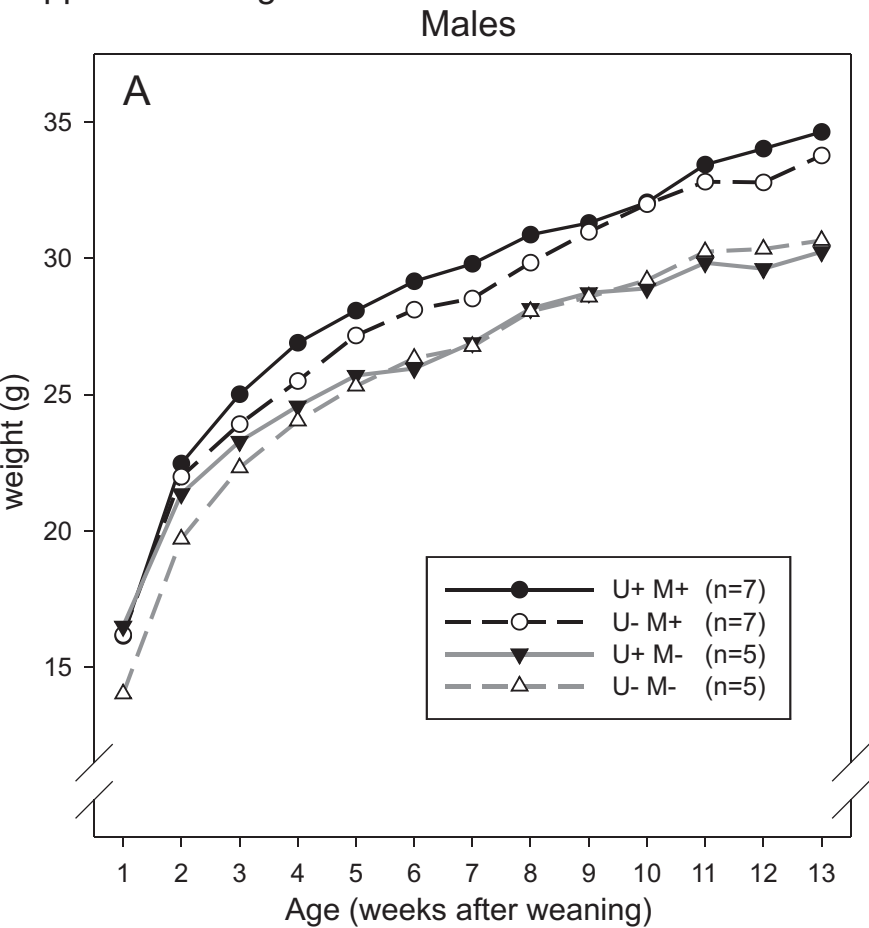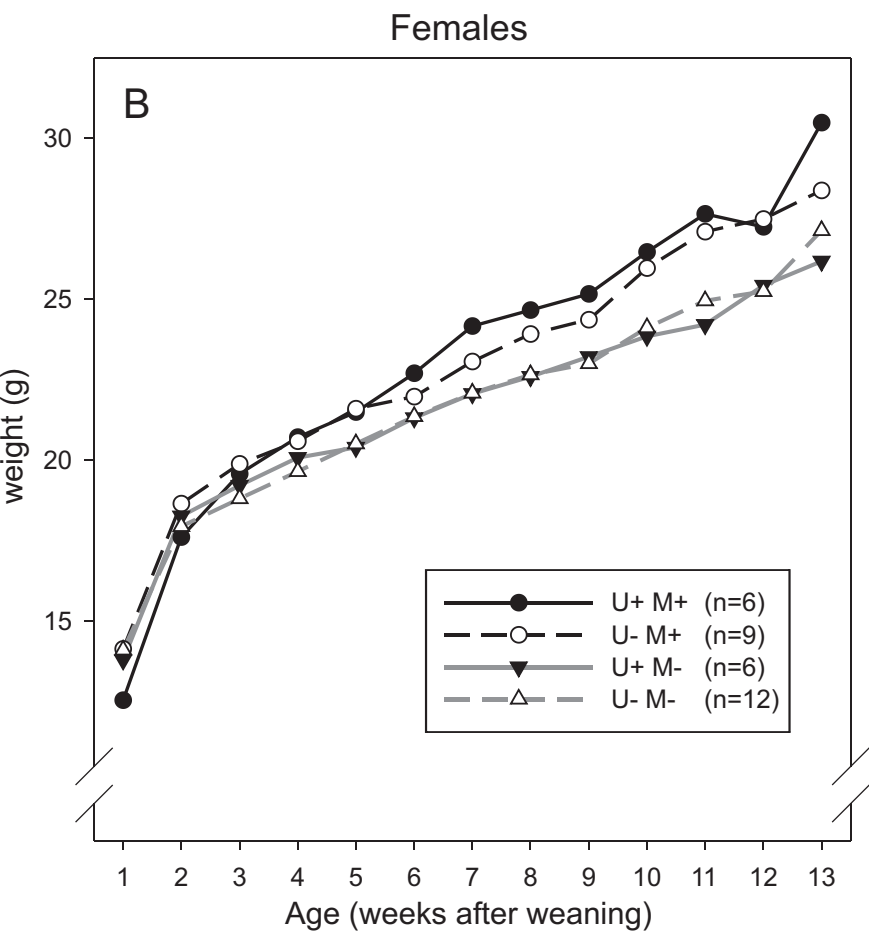

Supplement: Figure S1 — Post-weaning growth of mice with single and combined deficiency of uPARAP and MMP-2. The weight of a prospective cohort of mice was measured once a week after weaning for 13 weeks. The weight curves were compared using a two-way repeated measures ANOVA (see Methods). This analysis showed that the mice separated into two groups with U+M+ and U−M+ mice having a significantly faster weight gain than U+M− and U−M− mice. (PDF) [file pone.0071261.s001.pdf]
